# The roles of the outdoors and occupants in contributing to a potential pan-microbiome of the built environment: a review

**DOI:** 10.1186/s40168-016-0165-2

**Published:** 2016-05-24

**Authors:** Marcus H. Y. Leung, Patrick K. H. Lee

**Affiliations:** School of Energy and Environment, City University of Hong Kong, Tat Chee Avenue, Kowloon, B5423-AC1 Hong Kong

**Keywords:** Indoor built environments, Geography, Building designs, Outdoor air, Human microbiome, Pan-microbiome

## Abstract

Recent high-throughput sequencing technology has led to an expansion of knowledge regarding the microbial communities (microbiome) across various built environments (BEs). The microbiome of the BE is dependent upon building factors and conditions that govern how outdoor microbes enter and persist in the BE. Additionally, occupants are crucial in shaping the microbiome of the BE by releasing human-associated microorganisms and resuspending microbes on floors and surfaces. Therefore, both the outdoors and occupants act as major sources of microorganisms found in the BE. However, most characterizations of the microbiome of the BE have been conducted in the Western world. Notably, outdoor locations and population groups present geographical variations in outdoor and human microbiomes, respectively. Given the influences of the outdoor and human microbiomes on BE microbiology, and the geographical variations in outdoor and human microbiomes, it is likely that the microbiomes of BEs also vary by location. The summation of microbiomes between BEs contribute to a potential BE pan-microbiome, which will both consist of microbes that are ubiquitous in indoor environments around the world, and microbes that appear to be endemic to particular geographical locations. Importantly, the BE pan-microbiome can potentially question the global application of our current views on indoor microbiology. In this review, we first provide an assessment on the roles of building and occupant properties on shaping the microbiome of the BE. This is then followed by a description of geographical variations in the microbiomes of the outdoors and humans, the two main sources of microbes in BEs. We present evidence of differences in microbiomes of BEs around the world, demonstrating the existence of a global pan-microbiome of the BE that is larger than the microbiome of any single indoor environment. Finally, we discuss the significance of understanding the BE pan-microbiome and identifying universal and location-specific relationships between building and occupant characteristics and indoor microbiology. This review highlights the much needed efforts towards determining the pan-microbiome of the BE, thereby identifying general and location-specific links between the microbial communities of the outdoors, human, and BE ecosystems, ultimately improving the health, comfort, and productivity of occupants around the world.

**Electronic supplementary material:**

The online version of this article (doi:10.1186/s40168-016-0165-2) contains supplementary material, which is available to authorized users.

## Background

Recent advances in culture-independent, DNA sequencing (hereafter referred to as high-throughput sequencing, HTS) technology have led to unprecedented insights into the diverse repertoire of microorganisms (microbiome), including bacteria, fungi, viruses, and parasites, that are present in various environments. It is now appreciated that cultivation-based methods represent <0.001 % of the total microbial life truly present [1]. HTS has been used to characterize microbial communities at much greater depth and in different environments, highlighting the importance of microbial life in driving biological and biochemical processes in various ecosystems [2–5].

Of these different ecosystems, the microbiome of the indoor environment (or built environment, BE) has been investigated in detail over the past decade. Recent efforts to characterize microbial community of the BE are motivated by the fact that modern urbanites allocate approximately 90 % of their times indoors [6], making the BE a predominant habitat for humans in the developed world. In the developing world, trends towards urbanization and modernization will likely be concomitant with people spending more time indoors. Currently, over 70 % of the fastest growing urban centers are situated in developing countries [7]. In addition, approximately 70 % of the world’s population will live in urban areas by the year 2050 [8]. From a microbial exposure perspective, the majority of microorganisms encountered by most humans’ lifetime will therefore be those present in the BE, such as those in indoor air [9–11] and on various surfaces [12–14]. Interests in characterizing the microbiome of the BE are based on the importance of understanding associations between occupant health and microbial exposure in indoor settings. While exposure to specific allergens and pathogens within the BE can result in adverse health outcomes [15–17], studies have shown that the nature of microbial communities as a whole within the BE also affects health of occupants. For instance, exposure to greater repertoire of microorganisms, which for example can be mediated through pet ownership, may confer protection against the onset of respiratory conditions [18, 19], and reduced microbial diversities in indoor settings have been linked to the development of respiratory conditions including asthma [20, 21]. In addition, microbiome characterizations in public indoor spaces can provide valuable information regarding public health surveillance and the transmission of infectious agents [22–30]. Also, in a recent review, Hoisington et al*.* [31] propose that understanding the relationships between the microbiomes of the BE and its occupants can help improve the occupants’ mental well-being, as numerous bacterial and fungal species detected indoors have been documented to affect psychological health.

Given the increasing trend towards an indoor lifestyle, and the significance of the BE microbiome on human health, a greater understanding of the factors that shape microbial communities in the BE is imperative in improving occupant health and productivity [32]. Currently, microbiome studies involving HTS have been applied to a diverse array of indoor environments, including but not limited to residences [9, 12, 14, 21, 33–41], schools [11, 17, 42, 43], hospitals [22–25, 29, 30], public transports [26–28], aircrafts [44], shopping malls [45], fitness centers [46, 47], offices [43, 48, 49], and child-care facilities [50]. These studies demonstrate that HTS technology is a feasible and comprehensive tool to ultimately understand the BE microbiome with which the occupants interact. Importantly, these studies have demonstrated that the BE presents microbial communities distinct from other ecosystems and presents a unique assemblage ([22, 51, 52] and reviewed in [53]). Our recent knowledge regarding the microbial community of the BE is no longer limited to the types of microorganisms present, but also how various building and occupant characteristics alter the BE microbiome, and potentially occupant health. Therefore, rationales for characterizing microbial assemblages in the BE have become multifaceted and interdisciplinary in nature, combining elements of engineering, architecture, and microbiology. Also, regardless of the building types and locations in question, the outdoors and occupants have shown to be among the principal sources of microorganisms detected in BEs [9, 11–14, 36, 54]. Thus, various building and occupant characteristics and activities shape the microbial assemblage of the BE around the world, by influencing how outdoor- and human-associated microbes (unless otherwise indicated, microbes refer to bacteria and fungi in this review) are introduced into the indoors.

Despite the knowledge we have acquired regarding the roles of building and occupant characteristics on structuring the microbiome of the BE, the majority of these studies pertain to the Western world (Table 1 and Additional file 1). Conversely, studies characterizing the microbiomes of BEs in other geographical regions, where the majority of the world’s population are based, are limited (Table 1). Microbiome differences in local, regional, and continental scales have been documented across ecosystems [17, 55, 56]. Given that geographical clustering of microbial communities has been documented in the outdoors and humans (the two major sources of microorganisms in BEs, among others, recently reviewed in [57]), it is envisioned that such variations will create differences in microbiomes of BEs globally, because of the fundamental roles of the outdoors and occupants have on the BE microbiome. Ultimately, this collection of microbiomes contributes to a global BE pan-microbiome, which comprises of microorganisms that are detected in BEs across continental scales, as well as endemic microbes present in specific geographical locations. Understanding the pan-microbiome, which is greater than the microbiome of any single BE, has significant importance. For example, microbiome analyses of the BEs around the world may potentially uncover novel indoor microbial members that are specific to particular geographical locations. As the majority of the population lives outside of the Western hemisphere, it is uncertain whether our current insight on microbial community of the BE can be applied across areas with different living conditions, properties, and outdoor and occupant source microbiomes [58, 59]. In order to devise globally representative and location-specific strategies for improving indoor qualities within BEs, the differences in the microbiomes of the outdoors and occupants, acting as sources of microorganisms detected in the BE, and how that influence the pan-microbiome of the BE, need to be considered and appreciated.

**Table 1:** Countries, and their populations, where HTS-based microbiome works of the BE have been conducted. A total of 72 studies involving HTS are included based on search terms “indoor microbiome,” “built environment microbiome,” “built environment microbiota,” and “indoor microbial community” on the NCBI database and Google Scholar (reference list available as Additional file 1). All studies were funded by the corresponding countries where the samples were collected. According to the reference list compiled, over 90 % of the world’s population live in countries where microbiome data for the BE is unavailable

Sampling location	Region	Study count^a^	Population (million)^b^	Reference
Austria	Europe	3	8.5	[1–3]
Canada	North America	2	35.5	[4, 5]
Finland	Europe	2	5.5	[6, 7]
France	Europe	2	66.2	[8, 9]
Germany	Europe	1	80.9	[10]
Hong Kong	Asia	2	7.2	[11, 12]
International^c^	–	5	–	[13–17]
Singapore	Asia	1	5.5	[18]
South Korea	Asia	2	50.4	[19, 20]
Spain	Europe	1	46.4	[21]
Taiwan^b^	Asia	1	23.4	[22]
United States	North America	50	318.9	[23–72]
Others	–	0	6,612.0	

^a^List of references available as Additional file 1

^b^Except for Taiwan, figures based on 2014 data from The World Bank (http://data.worldbank.org/indicator/SP.POP.TOTL). Taiwan figures based on 2014 data from IndexMundi (http://www.indexmundi.com/taiwan/demographics_profile.html)

^c^Studies involving comparison of the microbiome of the BE in multiple locations and countries. All four international studies were funded by the USA

The key purposes of this review are to discuss the three fundamental and universal factors (mode of ventilation, building design, and occupancy) that shape the microbiome of the BE irrespective of geography, followed by a review of the evidence showing geography-based differences in the microbiomes of the outdoors, occupants, and the BE. We conclude the review with a description of the challenges and future directions in BE microbiome research, paying attention to the need to identify both universal and location-specific relationships between building and occupant characteristics and the microbiome of the indoor environment. Through this review, we encourage a greater focus on the characterization of microbiomes in indoor environments in geographically distinct locations, leading to the discovery of a BE pan-microbiome, which will ultimately assist in improving occupant health and comfort in BEs around the world.

## Microbiome of the BE before the era of HTS

Although HTS-based studies have drastically empowered scientists in understanding the shaping factors of the microbiome of the BE, interests in determining the microbial repertoire of the BE arose before the advent of HTS. These pre-HTS studies pave way for dedicated efforts in determining the relationships between environmental and anthropogenic factors and properties that help shape the microbiome of the BE.

Through earlier culture-dependent studies [60–64], Gram-positive bacteria, including human-associated *Staphylococcus*, *Micrococcus*, and soil dweller *Bacillus*, and fungi including *Penicillium*, *Aspergillus*, and *Cladosporium*, are among the most commonly cultured microorganisms from the BE. The roles of the outdoor environment and occupants in shaping the microbiome of the BE are evident, when Pasanen et al*.* [61] demonstrated that BEs in farmlands differ in their microbiomes compared to urban BEs, potentially mediated by occupants introducing microorganisms associated with rural terrains. Additionally, the community of cultivable microbes may differ depending on the building type and design [64]. Therefore, these studies already demonstrate the importance of building design, the outdoors and occupants in shaping the microbiome of the BE. Culture-based studies also highlight the importance of determining the microbiome of the BE in terms of occupant and public health, as potentially pathogenic microorganisms can be cultured in various indoor environments [65, 66].

However, our insight into the microbial diversity of the BE at that time was limited to microbes that are cultivable under specific, stringent, and artificial laboratory conditions. Indeed, in studies that employ both culture and clone library-based sequencing, the bacterial and fungal diversities of the BE detected through sequencing greatly surpasses that of culture methods, impacting researchers to reconsider their views on the true breadth of microbial life of the indoors [15, 62, 67, 68]. Specifically, the enhanced diversity information obtained from clone library sequencing enables more in-depth analyses of how the outdoor environment and occupant activities shape the indoor microbiome [62, 69, 70], as well as how potential pathogens detected within indoor spaces may pose health risks for its occupants [15, 71]. Of interest, Täubel and colleagues [69] demonstrate that the inclusion of multiple household samples increases the total number of taxa detected, indicating that the idea of a BE pan-microbiome was appreciated even prior to when HTS became commonplace in indoor microbiome studies.

## Fundamental factors affecting BE microbiomes

### Mode of ventilation and building design

HTS studies analyzing the effect of ventilation modes of various BEs [10, 11, 22, 52] show that, in addition to the distinction of microbial communities in the BE compared to that of the outdoors, mechanically ventilated rooms also present different microbiomes to that of naturally ventilated indoor spaces. Naturally ventilated rooms tend to contain more similar microbiomes from adjacent outdoor air compared to that of mechanically ventilated rooms [11, 22]. Rooms with natural ventilation are perhaps more likely to facilitate microbes from the outdoors to enter [10, 11, 27]. Consistent with this, the abundances of microbes with outdoor origins have been shown to be greater in dust samples from naturally ventilated rooms [43]. At the same time, depending on the building type, mechanical ventilation can include filters, preventing some of the outdoor microbes and particulates from traveling into the BE [11, 22]. In addition to facilitating or impeding outdoor microbes from entering BEs, ventilation is likely to affect additional environmental parameters, including indoor temperature, humidity, airflow rates, and carbon dioxide levels. These parameters have also been reported to affect indoor microbial community compositions and may select for the survival of specific microbial taxa [22, 26, 33, 43]. Therefore, the type of ventilation mode selected for a particular BE is a major player in shaping the microbial community composition of the BE, by directly affecting how outdoor microbes enter into BE spaces, at the same time modulating environmental and selective properties within the BE.

In addition to ventilation strategy, building designs, such as room type, floor area, floor level, and spatial relationships with neighboring indoor spaces, can affect the microbiology of the indoor environment. Building design and architectural elements possibly shape the microbiome of the BE by mediating how air and microbes within the air are circulated within the BE. In offices and classrooms, Kembel and colleagues [43] report microbiome variations between rooms that differ in their accessibilities to adjacent indoor spaces (hallways with connections to a large number of other rooms have distinct microbiome to that of restrooms and rooms with fewer connections). Similarly, Adams et al*.* [52] document the variations in microbial communities between different room types. Specifically, outdoor-associated bacteria decrease in abundances within the indoor environment as one moves away from the outdoors into more interior parts of the BE. Moreover, different rooms within buildings may present distinct microbial communities and differ in microbial diversity [9, 12, 24, 43, 52]. For example, restrooms may present distinct microbiomes compared to living rooms and kitchens in residential settings [9, 12, 52], whereas microbiomes between rooms within other types of BEs may also vary [24, 43]. This is perhaps partly explained by variations in architectural strategies and floor plan adopted between space types to maximize occupant efficiency and functionality (for example, hallways with connection to other indoor spaces compared to restrooms with low connectivity to other indoor rooms [43]).

Indoor environments such as metropolitan subway systems provide a useful model for exploring the relationship between BE and outdoor microbiomes, and how this is shaped by architectural and usage variation. For example, subways around the world differ in a number of properties, including ventilation at stations and trains, platform location (indoor or outdoor, aboveground or underground), and presence of floor-to-ceiling safety screen doors between platforms and trains. These properties together can govern how air is introduced and circulated inside the subway environment [72]. Robertson et al*.* [27] characterized the air microbiome of the New York City metro network, showing efficient air mixing and hence insignificant variation in community compositions between subway and outdoor air. This is likely due to the piston effect of carriages in the absence of mechanical ventilation. In contrast, in the Hong Kong (HK) subway network [26], where mechanical ventilation is adopted and safety screen doors are installed where possible, a higher bioaerosol microbial diversity in outdoor air compared to that of the subway suggests that complete air mixing does not take place. Furthermore, according to their architectural relationship to the outdoors, different aboveground or underground subway lines show variations in microbial assemblages, and that the adjacent outdoor air is likely to be a major microbiome source for each subway line. Taken together, the works described above demonstrate the interplay between ventilation mode, architectural choices, and microbial community of the BE, governing how air from the outside is introduced into and circulated in the indoors. Further works in the BE microbiome research community can be focused on the temporal aspects of building or room-associated microbiome variations, so as to determine whether the observed differences in the microbial communities between BEs are temporally stable [73]. However, building design and room types also impinge on the density and activity of occupants, which, as we describe below, act as another major force configuring the microbiome within the BE.

### Human microbiome and occupancy

Given that humans shed approximately 10^7^ bacteria per person per hour in indoor settings [74], and that humans spend most of their times indoors, it should come as no surprise that human presence and activities contribute to the BE microbiomes. Indeed, to varying extents, microbiome studies of the BE all indicate the influence of occupants on shaping indoor microbial assemblages. Studies using source tracking and taxonomic approaches generally agree that microbiomes from indoor air and surfaces contain microorganisms predominantly associated with the human skin, with the human gut, oral, and urogenital microbiomes acting as additional sources of microbial communities in the BE [12, 26, 28, 36, 39, 42, 47, 52, 75]. Microbiome characterizations conducted in residences demonstrate that host- and household-specific microbial communities can be detected, and at times these household microbiomes resemble that of the occupants [9, 10, 12, 36, 52]. Similarly, a recent chamber study reveals that occupants tend to rapidly generate a “microbial cloud,” resulting in a change in the microbiome of the adjacent air compared to a vacant but otherwise identical space [76]. Interestingly, this microbial cloud is unique to each occupant at a community and a species or strain level. Similar to indoor bioaerosols, the rapid filling of a personal microbiome by its occupants also occurs along indoor floors and surfaces, demonstrating that occupants can quickly leave their microbiome fingerprint onto different ecosystems within the BE [36, 47, 77, 78]. Such observations have even led to the recent prospects of analyzing the microbiomes of humans and its surrounding environments for personal identification and forensic purposes [77, 79, 80].

Human contact with indoor surfaces is a way in which microbiome of the occupant affects the microbiome of indoor surfaces [36, 47, 80]. In addition to desquamation, skin-associated microbes can be transferred onto indoor surfaces and floors following physical contact. Studies investigating BE surfaces indicate both the frequency [14], as well as the nature of human contact (e.g. whether surface contact mediated with skin on handles and grips, or mediated with shoes on floor and carpets, or release of gut-associated microbes in washrooms) [13, 42, 43, 46, 47, 75], are associated with variations in microbiomes of different indoor areas and even on different surfaces within a single BE or room. Therefore, different sub-microbiomes may exist within an indoor space, depending on the types of contacts it has with the human body. Furthermore, longitudinal analysis reveals more extensive community dynamics on surfaces frequented by human contact, suggesting that surfaces in public areas (e.g. fitness centers, airplanes, public transports, etc.), where they are likely to come into contact with more people, may also experience greater temporal variations in their microbial communities [47] compared to a private BE (residential unit). These observations in general underscore how occupant contact affects microbiomes of indoor surfaces in various ways, and that understanding the types of activities that occupants engage in will aid in predicting the resulting microbial communities in different BEs.

In addition to direct contact, a number of works have shown that human occupancy and movements also affect indoor microbiomes. Re-suspension of settled dust particles via movement of the occupants has been demonstrated to be a source of indoor microbial particle emissions [17, 48, 54, 81]. Kembel et al*.* [43] show that indoor spaces with a high human occupancy and traffic (such as hallways) present distinct microbial communities compared to spaces with a lower human occupancy and traffic. A number of other works demonstrate that occupancy is associated with increased particle mass, microbial loads, concentration, and diversity and abundance of human-associated microbes in indoor air [11, 41, 54, 73, 81]. In addition, human and domesticated animal movements and activities inside and outside of the BE can also affect the indoor microbiome, by introducing exogenous microbial members into the BE [48, 51, 82, 83]. Therefore, the roles of human on shaping the microbiome of the BE are not limited to the human microbiome but also by occupant activities, movements, and their relationships with the immediate environments.

## Geographical differences in the sources of major BE microbiomes

### Geographical differences in the outdoor microbiomes

Given the importance of the outdoor environment as a microbial source for the microbial assemblage of the BE, changes that affect outdoor microbial community will potentially influence the microbiome of the nearby BE. The outdoor microbiome itself is a conglomerate of microorganisms from nearby soil, plant, and aquatic environments, which are renowned for their extensive microbial diversities [2, 3, 84]. Moreover, microbial compositions of these source environments are subjected to wide fluctuations of environmental conditions, facilitating the survival and growth of different microorganisms [85]. As a result, variations in the adjacent terrain will lead to different outdoor air community structures depending on location [86–88]. Consistent with this, differences in microbiomes of the atmosphere have been associated with variations in land use types. Bowers et al*.* [55] compare the microbiomes of the air above agricultural, suburban, and forest areas, revealing strong terrain-based clustering of microbiomes in the near-surface atmosphere, driven by changes in the abundances of bacterial taxa indigenous of local surroundings.

In addition to geography and landform, local weather condition is another factor in structuring the microbial community of the outdoor air. Meteorological and climatic conditions affect the microbiome by governing how microbes are aerosolized, transported, and dispersed (reviewed in [89]). Specifically, weather condition potentially dictates how different sources of microbiomes contribute to the microbial community of the outdoors. For example, dryer and warmer conditions may encourage dispersal of soil- and plant-associated microbes following desiccation, while colder weather conditions may be associated with presence of cold-tolerant and ice-associated microorganisms in the air [90, 91]. Also, seasonal differences may be associated with the import of air masses from different terrains, contributing to variations in microbial communities of the outdoors. Woo and colleagues [92] demonstrate that in HK, air masses in the summer originate from the aquatic south, while air masses come from the terrestrial north during the winter. Interestingly, this seasonal air mass source difference is associated with changes in the abundances of marine and soil-associated microorganisms in the outdoor air.

Urbanization may have a negligible role in shaping outdoor microbial communities, as shown in the aforementioned HK study [92], but a separate US study demonstrates the effect of urbanization on dampening the variations in the microbiomes of different areas, such that the microbiome dissimilarities between cities are less than that of samples across rural areas [93]. Intriguingly, with a large enough sample size and the appropriate statistical predictive tools, the geographical location of a sample can be identified within a 200-km radius based on its microbial community [94]. Taken together, these studies corroborate the endemic nature of the microbiome of the outdoors and highlight the importance of nearby landforms, as well as environmental and meteorological conditions, in understanding outdoor bioaerosol compositions across geography. Importantly, information about variations in microbiomes between locations can be applied to design predictive and computational tools useful for microbial ecologists. Such tools will ultimately help identify patterns of microbial community changes associated with geography, terrain and developments, and climatic conditions.

### Differences in human microbiomes based on geographical location

The majority of the human microbiome studies to date pertain to Western subjects. Yet, global citizens are of different geographical origins, and our current knowledge about the human microbiome, and its relationships with physiologies, health, and diseases, may not reflect on population groups with discrepant lifestyles and environmental exposures. Until the last 5 years, population group differences on the human microbiome appear to have been overlooked [95]. However, recent analyses of microbial communities of population groups demonstrate that the global human microbiome community exists as a human pan-microbiome, larger than that of any single person or population group, and that lifestyle changes associated with modernization over time has led to a change in microbial communities compared to our ancestors [59, 96, 97].

Given that human occupancy and activities are major shaping forces of the microbiome of the BE, and the human microbiome is a main source for the microbiome of indoor spaces, geography-based variations in human microbial communities will also likely contribute to a pan-microbiome of a global BE. Therefore, an appreciation for the human pan-microbiome is essential in understanding the pan-microbiome of the BE. The following section highlights the key research works dedicated to the comparison of the human microbiomes (mainly the gut, oral, and skin microbiomes, as these are the key sources of humans’ influence in the microbiome of the BE) between different population groups. We stress that microbiome differences between populations do not necessarily equate variations in microbial communities between different ethnic or racial groups. It is unlikely that ethnicity and race inherently drive microbiome differences. Hence, these broad terms should not be considered predictor variables in understanding the relationships between microbiomes of the occupants and that of the BE [96, 98]. Instead, the terms ethnicity and race should be treated as starting points to uncover environmental exposures and lifestyle choices, potentially associated with ethnicity and race, that may play more direct roles in shaping the microbiomes of the occupants.

#### Gut microbiome (GM)

GM can spatially and temporally affect the microbial communities of indoor areas including washrooms, where usually relatively higher proportions of gut-associated microorganisms can be found [13, 43, 75]. In a study comparing GMs between cohorts, De Filippo et al*.* [99] attribute dietary differences as a main factor for the observed discrepancies in GMs between children in rural Burkina Faso and those in urban Italy. Their study also shows greater microbial richness and diversity in the guts of rural subjects, an observation recapitulated in later studies [58, 59, 100–102]. Researchers hypothesize that the populations of different geographical groups will adopt different dietary habits that vary in vegetable, fiber, starch and simple sugars, dairy products, and salt intakes. Subsequently, these variations will select for distinctive microbial populations and their specialized metabolic needs [103–106]. The greater diversity seen in rural cohorts around the world is also consistent with the hypothesis that westernization, industrialization, and urbanization lead to a reduction in the diversity of the GM. The loss of microbial diversity has direct health consequences, as urbanites may be more susceptible to diseases due to the loss of potentially beneficial microbes present in ancestral and tribal communities [58, 99, 102, 107]. Alternatively, in addition to the comparisons of different population groups, David et al*.* [108] reveal the changes in GMs of one individual, who relocated from an urban Western setting to a developing nation and subsequently adopted new dietary habits. That change in lifestyle and environmental exposure coincides with a change in GM to a different state that is reversed upon return to the subject’s place of origin, providing additional evidence that lifestyle changes associated with geography can affect microbial assemblages of the gut.

#### Oral microbiome (OM)

Interests in deciphering inter-cohort OM differences originally stem from variations in the prevalence of oral diseases between populations [109]. A study including subjects from 12 towns globally shows that population groups by location have significant differences in the abundances of specific genera within their saliva [110]. One subsequent study, sampling multiple sites within the oral cavities (supragingival, subgingival, and saliva) of different individuals in the US, shows population-unique species (differences in community memberships between populations) and differences between populations in the abundance of shared microbial members (differences in community composition between populations) [111]. While the majority of studies are limited to characterization of bacterial communities, Ghannoum et al*.* [112] examined fungal communities within oral rinses from White, African-American, Asian, and Native American individuals within Cleveland, Ohio, showing population-based as well as gender-based factors in shaping the fungal communities. Despite the observations documented by these studies, they suffer from either a lack of information regarding dietary habits and other lifestyle characteristics [110–112] or from a small number of study subjects [110, 112]. Furthermore, one study [110] employs Sanger sequencing now considered to be low throughput; hence, the true population-based variations in OM may be underestimated. In general, population group-focused analyses on OM variations are limited, and more in-depth investigations on the potential roles of different population groups in shaping OM changes will be necessary in the future. In addition, while studies indicate the oral microbiome as a potential source of microbial community of the BE [12, 48, 54], works pertaining to the transfer of microbes from the oral cavity to indoor spaces, similar to that demonstrated for skin microbes [36], will enhance our understanding on how the OM contributes to the microbial assemblage of the BE.

#### Skin microbiome (SM)

The skin is the largest human organ, and its microbiome generally has the most direct relationship with the immediate environment including the BE (described above). Daily activities and the external surroundings will have a prominent role in shaping a subject’s SM, as their activities, lifestyles, and the environments they are exposed to can potentially be inferred by the microbial populations present on various skin sites [97, 113, 114]. Skin physiologies have been shown to both differ by population group and affect the SM ([115, 116] and reviewed in [117]). In addition to host physiological properties, anthropogenic characteristics, such as gender, age, handedness, personal hygiene, and lifestyles, have all been shown to affect SM [96, 113, 118–120]. Our comparison of SMs between urban and rural populations reveals the expansion of a global cutaneous pan-microbiome [96]. Also, we detected a relatively high abundance of *Enhydrobacter* in Hong Kong individuals consistent with previous studies conducted in China [113, 121]. This genus is previously known to adopt an aquatic habitat and was only recently detected in individuals and BEs [47, 96, 121–123]. Hence, the detection of *Enhydrobacter* in Chinese individuals signifies that some microbes, previously known to be of environmental origins, may in fact be common colonizers of the human host in another part of the world.

A multi-site (including skin) analysis conducted by the Human Microbiome Project, using metagenomic analysis, examined the metabolic potential of microbial communities between individuals and population groups [4]. They show differences in the abundances of *Pseudomonadales* in the population groups analyzed. There was, however, no mention of population-based differences in functional potential as inferred by metagenomic analysis. Future works employing metagenomic sequencing on skin samples between populations will further enhance our understanding of how population and environmental parameters affect both the microbial populations and their metabolic potentials.

## Multiple BEs make up the BE pan-microbiome

**Fig. 1:**
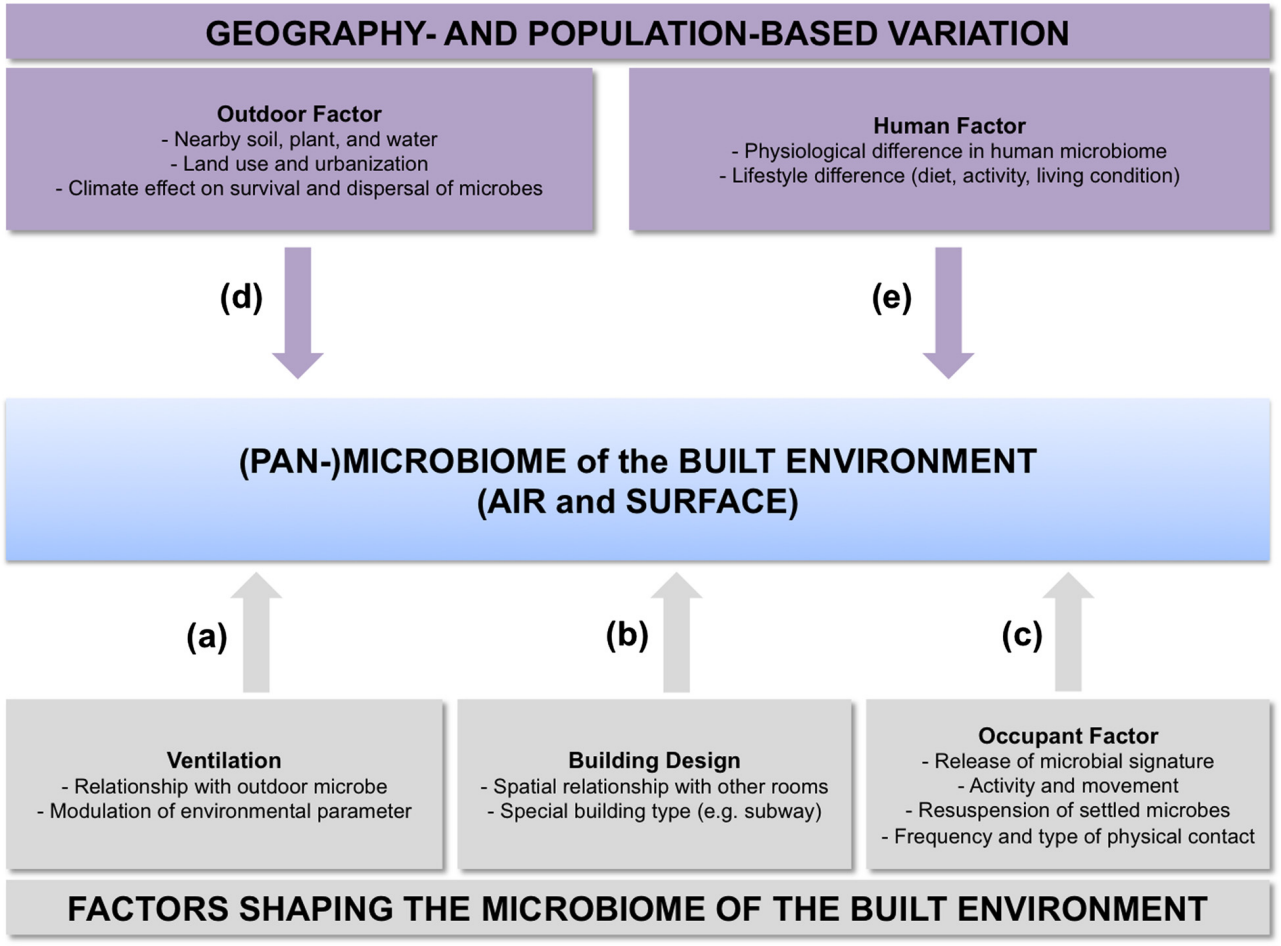
The pan-microbiome of the built environment (BE) is influenced by various factors. **a** Mode of ventilation within the BE facilitates both the introduction (natural ventilation) and the limit (mechanical ventilation) of outdoor microbes into the BE. The choice of ventilation mode also modulates environmental parameters including temperature, humidity, airflow, and carbon dioxide levels, affecting the survival and growth of specific microbes in the BE. **b** Building design affects the microbiome of the BE by the spatial relationships between different spaces within the BE, thereby influencing the flow of microorganisms within the indoor space. Also, special building types, such as screen doors and aboveground/underground rail lines associated with subways, may be associated with changes in the microbiome of the subway BE. **c** Humans in the BE release occupant-associated microbiomes by involuntary and voluntary means associated with physiology and activities. Occupant movements also re-suspend particles and microbes from surfaces and floors. Components **a**, **b**, and **c** therefore describe general factors shaping the microbiome of the BE (*grey boxes*). However, **d** outdoor sources of microbiomes may differ depending on the geographical location, as adjacent soil, plant, and water environments, land use, and level of urbanization will affect the microbial community composition of the immediate outdoors. Also, geography-based climate variations will also shape the microbiome of the outdoors, by affecting the survival and growth of certain microbes, and also influencing dispersal of microorganisms through outdoor spaces. **e** Population-based variations in microbiomes of human gut, oral cavity, and skin have been documented. Furthermore, lifestyle differences such as diet, activities, and living conditions will also affect what human-associated microbes are emitted into the BE. As a result, components **d** and **e** are major forces (*purple boxes*) that contribute to a global pan-microbiome of the BE, which is greater than the microbiome of any single BE

The effects of terrain, landforms, and climate, coupled with human physiological and anthropogenic properties, contribute to differences in the microbial compositions of the outdoors and occupants, two of the most important BE microbiome sources, around the world (Fig. 1). As the outdoors and occupants act as two predominant channels for introducing and emitting microorganisms into the indoors, it is anticipated that BEs around the world present distinctions in their microbial assemblages. Indeed, a number of studies have reported geographical variability in the microbial assemblage of the BE [10, 17, 49, 51, 56]. As we discuss below, we have only begun to dissect the extensive differences in the microbiome of the BE across geography, most of which are focused on the Western world.

Assessments of fungal communities in BEs across continents demonstrate that geography, rather than building design, best explains differences in microbial communities of the indoors, with human occupancy being another strong factor in shaping fungal communities of the indoors depending on occupant density [17, 56]. The influence of geography on fungal communities reinforces the role of outdoor environments in shaping the microbial community of the BE. Amend et al*.* [56] postulate that a combination of environmental selection and dispersal limitation governs the observed biogeography patterns in the BE, and that the relative strengths of the two factors differ depending on the taxa [56, 124]. Also, in the studies of Adams et al*.* [10, 52] investigating air microbiomes in residences within a housing complex, a positive correlation between similarities in microbial communities and geographical distances is detected, providing support that dispersal limitation within the BE also occurs on a local scale. On the other hand, bacterial communities in the BE are more likely to be associated with occupant characteristics and lifestyles [51]. Nonetheless, the role of geography can potentially alter bacterial communities in the BE through differences in human microbiomes, which in turn determine the microbes that are emitted into the indoor space. For example, the genus *Enhydrobacter*, which appears to be more abundant on the skin of Chinese individuals [96, 121], is also among the most abundant genera in air and on surfaces of various BEs throughout HK [9, 26]. Also, variations in indoor conditions that are associated with geography, such as occupant density and area size, may affect how microbes of different sources of the human microbiome (gut, mouth, skin) are released into the BE. For instance, Wilkins et al. [9] demonstrates little effect of occupants’ personal microbiomes in shaping microbial communities of the residential air, which contrasts from the American study of Meadow et al. [76], but consistent with other American studies [11, 48] that characterize different BEs. In the HK study [9], the gut and oral microbiomes may play greater roles as sources to the microbial communities of the residences. Also, given the high abundance of skin-associated bacteria in the outdoor air in HK (perhaps more so than in the USA [48, 81]) [26], the microorganisms detected in the residential air ecosystem in HK may originate from the outdoors.

**Fig. 2:**
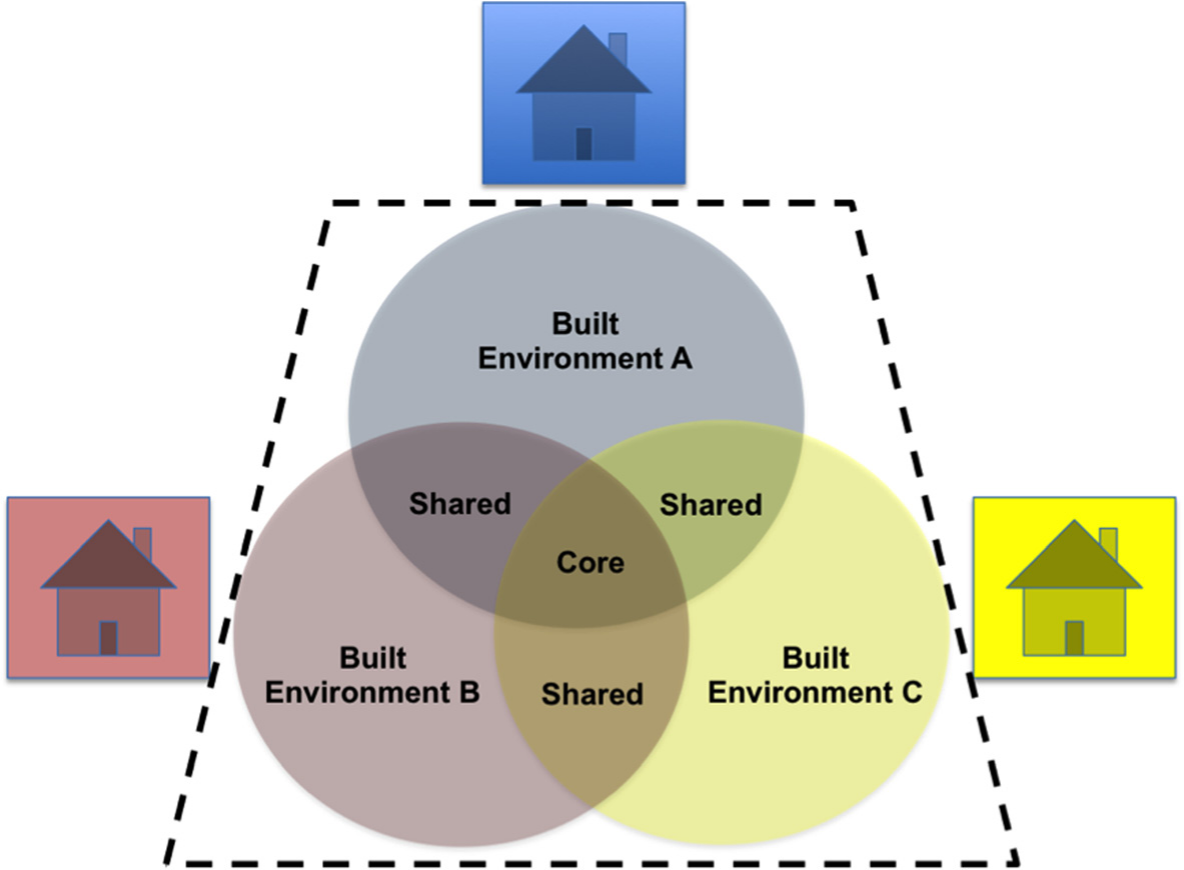
Different BEs constitute the pan-microbiome of the BE. Comparison of the microbiomes of multiple BEs will reveal taxa (of any taxonomic rank) that are detected in all the BEs, representing the core microbiome (overlapping region in the center). In addition, taxa that are shared between a subset of the BEs will be detected (overlapping area between any two BEs in the figure). Taxa that are specific or unique to a particular BE will also be detected (non-overlapping area). Taxa that are location-unique will contribute to expanding the pan-microbiome of the BE (trapezoid area within black dotted line) and may provide additional information concerning the relationships between building designs, the outdoor and occupant sources, and the microbiome of the specific BE

Despite the current limited knowledge regarding geographical differences in indoor microbiology, the works described above have begun to allow us to appreciate that the microbiomes of different BEs consolidate to form a pan-microbiome pool that is larger than the microbiome of any single indoor environment. Also, one can deduce the nature of this pan-microbiome pool (Fig. 2). A BE pan-microbiome first contains a collection of core microorganisms that are prevalent in BEs across all or most geographical locales. Given the roles of outdoors and humans in general on the microbial communities of the BE, core taxa within different BEs will include those commonly detected across the outdoors and the humans [43, 47, 75, 125]. The size of the core microbiome, here regarded as the number of taxa included, may depend on the locations [47], the numbers [56], and the types [9, 125] of BEs considered. Core bacteria common to different BEs may include those frequently detected on humans (for example, *Micrococcus*, *Acinetobacter*, and *Corynebacterium*) but may also include members of environmental origins (phylotypes of *Rhizobiales*, *Sphingobacteriales*, and *Sphingomonadales*) [9, 43, 125]. In addition to the core microbiome, the majority of the diversity seen across a pan-microbiome will potentially belong to taxa that are detected in subsets of the BEs considered. These unique (or distributed) taxa may represent a large proportion of OTUs within a pan-microbiome but may not necessarily represent a large proportion of total sequences [43, 125]. Such members can be identified through multi-study comparison of BE microbiome works using non-weighted community analyses or by taxonomic comparison between studies [26, 77]. For example, the taxonomic comparison of HK and New York City subway networks reveal that *Arthrobacter*, *Psychrobacter*, and *Enhydrobacter* may be considered distributed bacterial genera [26]. These distributed and endemic taxa can act as drivers of variations in microbiomes across different indoor environments, as they possibly originate from the microbial communities of nearby outdoor and human sources that are influenced by the many geography-associated factors discussed above (terrain, environmental factors, as well as occupant physiology and lifestyles). Following the dispersal from these source environments, various indoor conditions and building parameters that are unique to the BE will select for microbial members that can survive in a particular indoor environment [10, 53]. Indoor environmental conditions and specific indoor surfaces (such as metallic surfaces) may be associated with variations in the relative abundance of specific microbes [14, 26, 40, 126], while longitudinal studies shed light into identifying microbial members that are transient colonizers of the BE, versus those that may be capable of surviving and persisting within the BE [73, 75]. Taken together, the different outdoor, occupant, and indoor characteristics will ultimately shape the microbiome of a particular BE, and an assessment of microbiomes of a group of BEs will contribute to the pan-microbiome of the global BE.

Understanding the pan-microbiome of the BE has clinical significance. Microbial agents detected in BEs are associated with conditions such as asthma, which affects approximately 300 million individuals around the world [127]. In addition, Hanski and colleagues [128] describe the complex interactive nature between environmental biodiversity, occupant microbiomes, and immune response. Children with atopic epithelial conditions are directly associated with reduced environmental biodiversity in their nearby outdoor environments and lowered microbial diversity of skin microbial colonizers capable of eliciting anti-inflammatory responses. Therefore, it is essential to characterize the distribution of microorganisms across global BEs, as variations in the BE microbiome across the globe, and occupant exposure to allergens and other microbes in BEs, may in part explain geographical variations in the prevalence of allergenic and autoimmune diseases [17, 129]. Also, it has been appreciated that microbes in the BE do not exist and survive in solitude. Rather, microbes co-exist and potentially interact through polymicrobial communities that can alter their physiology, ecology, and virulence [130, 131]. If the microbiomes between BEs are different across geography, it is probable that the nature of potential interactions between microbes within the communities also differ between BEs. Therefore, greater understanding of variations in the microbiome of the BE in different locations may also shed insight into potential geographical differences in microbial interactions. Clearly, additional work is vital to characterize microbial populations and their interactions in BEs across the globe, evaluate how they are shaped by different building strategies and occupant characteristics, ascertain whether these properties are representative outside of the study area, and determine how these observations correlate with occupant health and productivity.

## Future considerations

With the ever-increasing number of people around the world adopting an indoor lifestyle, the need for a global understanding of the relationships between various building, environmental, and occupant properties and microbial communities in BEs has never been greater. Clearly, a focus in understanding the microbial community of the BE outside the Western world is necessary, as the majority of global citizens live outside the Americas and Europe. While fundamental factors, such as modes of ventilation, building design, and occupant properties and activities discussed previously, may shape microbiomes of BEs around the world in similar manners, geographical differences in microbiomes of outdoor and occupant sources may drive community differences between global BEs and ultimately expand the BE pan-microbiome. Predictions about the nature of this community variation, such as how BEs in developing world may differ from that of the developed world, potentially require additional investigations dedicated to the outdoor and occupant microbiome on a global scale. In order for the different studies to be comparable, standardized metadata collection, especially that of environmental conditions, building designs, and occupant characteristics, are of paramount importance. Such efforts can be facilitated by adopting currently available guidelines, as demonstrated in recent studies [132, 133], or by participating in global initiatives that promote the dissemination of laboratory and computational expertise, tools, and integration of scientific data [134, 135]. For now, comparisons between BE microbiome reports are limited to studies where different sampling, laboratory, bioinformatics, and statistical methodologies are adopted, underscoring the difficulties in generalizing universal relationships between environmental, building, occupant characteristics, and the BE microbiome [26, 136]. Thus, future investigations of the microbiome of the BE around the world should make laboratory workflows and metadata collection consistent, as standardization will undoubtedly empower our ability to determine the shaping forces of microbial communities of the BE that are globally representative. Notably, Adams et al*.* advocate the collection of matched outdoor samples to microbiome works of the BE, further emphasizing the importance of understanding the local outdoor environment in shaping the microbiome of indoor spaces [136]. In addition, the use of accurate and consistent terminologies, which is currently a subject of relevance in microbiome research [137], will also benefit the comparison of results across studies.

Also, while HTS technology has been applied to many microbiome investigations of the indoor environment, a great majority of these studies examine the microbial repertoire of the community, without assessing for viability of the detected organisms. It has been reported that as high as 90 % of the total DNA detected in a BE via HTS may originate from non-viable cells [138]. Microorganisms collected in air and on indoor surfaces have been demonstrated to be viable [75, 131, 139], however HTS transcriptome analysis of the BE will provide a more in-depth and comprehensive evaluation of the metabolically active microbes in indoor spaces. HTS methods can be performed in conjunction with standard photo-reactive dye-based assays (e.g., propidium monoazide), to assess viable subcomponents of the detected microbiomes of the BE [138, 140]. Through this understanding, we will gain insight into the various building, environmental, and human properties that may act as selective forces. These selective forces, along with dispersal limitation, may ultimately play an important role in shaping location-specific microbial populations across different BEs, hence contributing to the BE pan-microbiome. Understanding these selective forces may also provide support that the indoor microbiome is not simply a residue of the microbiomes of the outdoor and human sources but one that is subjected to unique sets of selective conditions, shaping its unique indoor microbiome.

Microbiome research of the BE should also recognize that novel buildings, with new building design, will be constructed in the coming years in response to social and environmental issues. For example, the increased need for energy sustainability, a crucial topic of the twenty-first century, calls for indoor spaces with innovative architectural strategies to minimize energy consumption. Green and zero-carbon buildings (ZCBs) are among emerging types of BEs in the developed and developing world, employing novel building characteristics including ventilation, temperature, humidity, landscaping, and occupant density to minimize energy use [141]. Currently, there is no microbiome information regarding such BEs. However, given what is reviewed here, the building characteristics ZCBs try to modulate may also affect the microbiome of the ZCB environment. Therefore, additional works investigating ZCBs and other emerging BEs around the world are warranted. This knowledge will inform us on how new building designs help structure the BE microbial assemblage and whether geographical variations will be present in these emerging BEs across geography.

## Conclusions

For the past decade, the scientific community has been blessed with the wealth of knowledge about the microbial community structure of the BE, how various building and occupant characteristics structure the microbiology of indoor spaces, and how the outdoors and human act as sources for introducing microorganisms into the indoor environment. However, our current extensive knowledge about microbiome of the BE in the Western world is met with a relative paucity of microbiome data on indoor settings elsewhere. We are beginning to appreciate that the microbiomes of the outdoors and humans are shaped by geography and associated environmental and occupant factors. At the same time, recent studies reveal the geographical differences in microbiomes of BEs around the globe, contributing to a collective BE pan-microbiome. While the significance of the BE pan-microbiome has not been elucidated, the presence of a global BE pan-microbiome questions the universality of our current knowledge about indoor microbiology. Additional efforts are necessary in identifying general and location-specific relationships between building properties, occupant characteristics, and microbiomes of the BE across the globe. By investing our efforts into understanding the microbiology of indoor environments in locations where the majority of the world’s population resides, we can present universally relevant perspectives on methods to optimize indoor environments. By striving for a global awareness in indoor microbiology, the scientific community will play an invaluable part in improving the health, well-being, and productivity of occupants around the globe.

## Additional file

Additional file 1: List of references used for Table 1. (DOCX 111 kb)

## Abbreviations

BE: built environment
DNA: deoxyribonucleic acid
GM: gut microbiome/gut microbiota
HK: Hong Kong
HTS: high-throughput sequencing
OM: oral microbiome/microbiota
OTU: operational taxonomic unit
SM: skin microbiome/microbiota
US: United States
ZCB: zero-carbon building
