# Supplementary material for: The roles of the outdoors and occupants in contributing to a potential pan-microbiome of the built environment: a review
Source: Microbiome. 2016 May 24;4:21. doi: 10.1186/s40168-016-0165-2 (PMC4877933; doi:10.1186/s40168-016-0165-2)
Supplement: Additional file 1: — List of references used for Table 1. (DOCX 111 kb) [file 40168_2016_165_MOESM1_ESM.docx]

**Additional File 1. List of references used for Table 1.**

**Austria**

1. Mahnert A, Moissl-Eichinger C, Berg G. Microbiome interplay: plants alter microbial abundance and diversity within the built environment. Front Microbiol. 2015;6:887.

2. Mahnert A, Vaishampayan P, Probst AJ, Auerbach A, Moissl-Eichinger C, Venkateswaran K, et al. Cleanroom maintenance significantly reduces abundance but not diversity of indoor microbiomes. PLoS ONE. 2015;10:e0134848.

3. Oberauner L, Zachow C, Lackner S, Högenauer C, Smolle KH, Berg G. The ignored diversity: complex bacterial communities in intensive care units revealed by 16S pyrosequencing. Sci Rep. 2013;3:1413.

**Canada**

4. Konya T, Koster B, Maughan H, Escobar M, Azad MB, Guttman DS, et al. Associations between bacterial communities of house dust and infant gut. Environ Res. 2014;131:25–30.

5. Ross AA, Neufeld JD. Microbial biogeography of a university campus. Microbiome. 2015;3:66.

**Finland**

6. Hultman J, Rahkila R, Ali J, Rousu J, Björkroth KJ. Meat processing plant microbiome and contamination patterns of cold-tolerant bacteria causing food safety and spoilage risks in the manufacture of vacuum-packaged cooked sausages. Appl Environ Microbiol. 2015;81:7088–7097.

7. Pitkäranta M, Meklin T, Hyvärinen A, Nevalainen A, Paulin L, Auvinen P, et al. Molecular profiling of fungal communities in moisture damaged buildings before and after remediation - a comparison of culture-dependent and culture-independent methods. BMC Microbiol. 2011;11:235.

**France**

8. Gaüzère C, Moletta-Denat M, Blanquart H, Ferreira S, Moularat S, Godon JJ, et al. Stability of airborne microbes in the Louvre Museum over time. Indoor Air. 2014;24:29–40.

9. Gaüzère C, Godon JJ, Blanquart H, Ferreira S, Moularat S, Robine E, et al. “Core species” in three sources of indoor air belonging to the human micro-environment to the exclusion of outdoor air. Sci Total Environ. 2014;485–486:508–517.

**Germany**

10. Moissl-Eichinger C, Auerbach AK, Probst AJ, Mahnert A, Tom L, Piceno Y, et al. Quo vadis? Microbial profiling revealed strong effects of cleanroom maintenance and routes of contamination in indoor environments. Sci Rep. 2015;5:9156.

**Hong Kong**

11. Leung MHY, Wilkins D, Li EKT, Kong FKF, Lee PKH. Indoor-air microbiome in an urban subway network: diversity and dynamics. Appl Environ Microbiol. 2014;80:6760–6770.

12. Wilkins D, Leung MHY, Lee PKH. Indoor air bacterial communities in Hong Kong households assemble independently of occupant skin microbiomes. Environ Microbiol. 2015;doi:10.1111/1462–2920.12889.

**International/Multi-Country**

13. Amend AS, Seifert KA, Samson R, Bruns TD. Indoor fungal composition is geographically patterned and more diverse in temperate zones than in the tropics. Proc Natl Acad Sci. 2010;107:13748–13753.

14. Barberán A, Ladau J, Leff JW, Pollard KS, Menninger HL, Dunn RR, et al. Continental-scale distributions of dust-associated bacteria and fungi. Proc Natl Acad Sci. 2015;112:5756–5761.

15. Checinska A, Probst AJ, Vaishampayan P, White JR, Kumar D, Stepanov VG, et al. Microbiomes of the dust particles collected from the International Space Station and Spacecraft Assembly Facilities. Microbiome. 2015;3:50.

16. Yamamoto N, Hospodsky D, Dannemiller KC, Nazaroff WW, Peccia J. Indoor emissions as a primary source of airborne allergenic fungal particles in classrooms. Environ Sci Technol. 2015;49:5098–5106.

17. Adams RI, Tian Y, Taylor JW, Bruns TD, Hyvärinen A, Täubel M. Passive dust collectors for assessing airborne microbial material. Microbiome. 2015;3:46.

**Singapore**

18. Tringe SG, Zhang T, Liu X, Yu Y, Lee WH, Yap J, et al. The airborne metagenome in an indoor urban environment. PLoS ONE. 2008;3:e1862.

**South Korea**

19. Jeon YS, Chun J, Kim BS. Identification of household bacterial community and analysis of species shared with human microbiome. Curr Microbiol. 2013;67:557.

20. Shin SK, Kim J, Ha SM, Oh HS, Chun J, Sohn J, et al. Metagenomic insights into the bioaerosols in the indoor and outdoor environments of childcare facilities. PLoS ONE. 2015;10:e0126960.

**Spain**

21. Poza M, Gayoso C, Gómez MJ, Rumbo-Feal S, Tomás M, Aranda J, et al. Exploring bacterial diversity in hospital environments by GS-FLX titanium pyrosequencing. PLoS ONE. 2012;7:e44105.

**Taiwan**

22. Tang CY, Yiu SM, Kuo HY, Tan TS, Liao KH, Liu CC, et al. Application of 16S rRNA metagenomics to analyze bacterial communities at a respiratory care centre in Taiwan. Appl Microbiol Biotechnol. 2014;99:1–11.

**United States**

23. Adams RI, Bateman AC, Bik HM, Meadow JF. Microbiota of the indoor environment: a meta-analysis. Microbiome. 2015;3:49.

24. Adams RI, Miletto M, Taylor JW, Bruns TD. Dispersal in microbes: fungi in indoor air are dominated by outdoor air and show dispersal limitation at short distances. ISME J. 2013;7:1262–1273.

25. Adams RI, Miletto M, Taylor JW, Bruns TD. The diversity and distribution of fungi on residential surfaces. PLoS ONE. 2013;8:e78866.

26. Adams RI, Bhangar S, Pasut W, Arens EA, Taylor JW, Lindow SE, et al. Chamber bioaerosol study: outdoor air and human occupants as sources of indoor airborne microbes. PLoS ONE. 2015;10:e0128022.

27. Adams RI, Miletto M, Lindow SE, Taylor JW, Bruns TD. Airborne bacterial communities in residences: similarities and differences with Fungi. PLoS ONE. 2014;9:e91283.

28. Adams RI, Amend AS, Taylor JW, Bruns TD. A unique signal distorts the perception of species richness and composition in high-throughput sequencing surveys of microbial communities: a case study of fungi in indoor dust. Microb Ecol. 2013;66:735–741.

29. Afshinnekoo E, Meydan C, Chowdhury S, Jaroudi D, Boyer C, Bernstein N, et al. Geospatial resolution of human and bacterial diversity with city-scale metagenomics. Cell Syst. 2015;1:72–87.

30. Barberán A, Dunn RR, Reich BJ, Pacifici K, Laber EB, Menninger HL, et al. The ecology of microscopic life in household dust. Proc R Soc B. 2015;282:20151139.

31. Bokulich NA, Mills DA. Facility-specific “house” microbiome drives microbial landscapes of artisan cheesemaking plants. Appl Environ Microbiol. 2013;79:5214.

32. Bokulich NA, Mills DA, Underwood M. Surface microbes in the neonatal intensive care unit: changes with routine cleaning and over time. J Clin Microbiol. 2013;51:2617–2624.

33. Bokulich NA, Bergsveinson J, Ziola B, Mills DA. Mapping microbial ecosystems and spoilage-gene flow in breweries highlights patterns of contamination and resistance. eLife. 2015;4:e04634.

34. Bräuer SL, Vuono D, Carmichael MJ, Pepe-Ranney C, Strom A, Rabinowitz E, et al. Microbial sequencing analyses suggest the presence of a fecal veneer on indoor climbing wall holds. Curr Microbiol. 2014;69:681–689.

35. Brooks B, Firek BA, Miller CS, Sharon I, Thomas BC, Baker R, et al. Microbes in the neonatal intensive care unit resemble those found in the gut of premature infants. Microbiome. 2014;2:1.

36. Dannemiller KC, Gent JF, Leaderer BP, Peccia J. Influence of housing characteristics on bacterial and fungal communities in homes of asthmatic children. Indoor Air. 2015;doi:10.1111/ina.12205.

37. Dannemiller KC, Mendell MJ, Macher JM, Kumagai K, Bradman A, Holland N, et al. Next-generation DNA sequencing reveals that low fungal diversity in house dust is associated with childhood asthma development. Indoor Air. 2014;24:236–247.

38. Dunn RR, Fierer N, Henley JB, Leff JW, Menninger HL. Home life: factors structuring the bacterial diversity found within and between homes. PLoS ONE. 2013;8:e64133.

39. Emerson JB, Keady PB, Brewer TE, Clements N, Morgan EE, Awerbuch J, et al. Impacts of flood damage on airborne bacteria and fungi in homes after the 2013 Colorado Front Range flood. Environ Sci Technol. 2015;49:2675–2684.

40. Feazel LM, Baumgartner LK, Peterson KL, Frank DN, Harris JK, Pace NR. Opportunistic pathogens enriched in showerhead biofilms. Proc Natl Acad Sci. 2009;106:16393–16399.

41. Flores GE, Bates ST, Caporaso JG, Lauber CL, Leff JW, Knight R, et al. Diversity, distribution and sources of bacteria in residential kitchens. Environ Microbiol. 2013;15:588–596.

42. Flores GE, Bates ST, Knights D, Lauber CL, Stombaugh J, Knight R, et al. Microbial biogeography of public restroom surfaces. PLoS ONE. 2011;6:e28132.

43. Fouquier J, Schwartz T, Kelley ST. Rapid assemblage of diverse environmental fungal communities on public restroom floors. Indoor Air. 2015;doi: 10.1111/ina.12279.

44. Fujimura KE, Johnson CC, Ownby DR, Cox MJ, Brodie EL, Havstad SL, et al. Man’s best friend? The effect of pet ownership on house dust microbial communities. J Allergy Clin Immunol. 2010;126:410–412.e3.

45. Gibbons SM, Schwartz T, Fouquier J, Mitchell M, Sangwan N, Gilbert JA, et al. Ecological succession and viability of human-associated microbiota on restroom surfaces. Appl Environ Microbiol. 2015;81:765–773.

46. Hewitt KM, Gerba CP, Maxwell SL, Kelley ST. Bacterial abundance and diversity in three metropolitan areas. PLoS ONE. 2012;7:e37849.

47. Hoisington AJ, Maestre JP, King MD, Siegel JA, Kinney KA. Impact of sampler selection on the characterization of the indoor microbiome via high-throughput sequencing. Build Environ. 2014;80:274–282.

48. Hoisington A, Maestre JP, Siegel JA, Kinney KA. Exploring the microbiome of the built environment: A primer on four biological methods available to building professionals. HVACR Res. 2014;20:167–175.

49. Hoisington A, Maestre JP, Kinney KA, Siegel JA. Characterizing the bacterial communities in retail stores in the United States. Indoor Air. 2015; doi:10.1111/ina.12273.

50. Hong PY, Li X, Yang X, Shinkai T, Zhang Y, Wang X, et al. Monitoring airborne biotic contaminants in the indoor environment of pig and poultry confinement buildings. Environ Microbiol. 2012;14:1420–1431.

51. Hospodsky D, Qian J, Nazaroff WW, Yamamoto N, Bibby K, Rismani-Yazdi H, et al. Human occupancy as a source of indoor airborne bacteria. PLoS ONE. 2012;7:e34867.

52. Kembel SW, Meadow JF, O’Connor TK, Mhuireach G, Northcutt D, Kline J, et al. Architectural design drives the biogeography of indoor bacterial communities. PLoS ONE. 2014;9:e87093.

53. Kembel SW, Jones E, Kline J, Northcutt D, Stenson J, Womack AM, et al. Architectural design influences the diversity and structure of the built environment microbiome. ISME J. 2012;6:1469–1479.

54. Kettleson EM, Adhikari A, Vesper S, Coombs K, Indugula R, Reponen T. Key determinants of the fungal and bacterial microbiomes in homes. Environ Res. 2015;138:130–135.

55. Lax S, Smith DP, Hampton-Marcell J, Owens SM, Handley KM, Scott NM, et al. Longitudinal analysis of microbial interaction between humans and the indoor environment. Science. 2014;345:1048–1052.

56. Meadow JF, Altrichter AE, Kembel SW, Kline J, Mhuireach G, Moriyama M, et al. Indoor airborne bacterial communities are influenced by ventilation, occupancy, and outdoor air source. Indoor Air. 2014;24:41–48.

57. Meadow JF, Altrichter AE, Bateman AC, Stenson J, Brown G, Green JL, et al. Humans differ in their personal microbial cloud. PeerJ. 2015;3:e1258.

58. Meadow JF, Altrichter AE, Kembel SW, Moriyama M, O’Connor TK, Womack AM, et al. Bacterial communities on classroom surfaces vary with human contact. Microbiome. 2014;2:7.

59. Meadow JF, Altrichter AE, Green JL. Mobile phones carry the personal microbiome of their owners. PeerJ. 2014;2:e447.

60. Miletto M, Lindow SE. Relative and contextual contribution of different sources to the composition and abundance of indoor air bacteria in residences. Microbiome. 2015;3:61.

61. Mukherjee N, Dowd SE, Wise A, Kedia S, Vohra V, Banerjee P. Diversity of bacterial communities of fitness center surfaces in a U.S. metropolitan area. Int J Environ Res Public Health. 2014;11:12544–12561.

62. Nonnenmann MW, Coronado G, Thompson B, Griffith WC, Hanson JD, Vesper S, et al. Utilizing pyrosequencing and quantitative PCR to characterize fungal populations among house dust samples. J Environ Monit. 2012;14:2038–2043.

63. Pinto AJ, Schroeder J, Lunn M, Sloan W, Raskin L. Spatial-temporal survey and occupancy-abundance modeling to predict bacterial community dynamics in the drinking water microbiome. mBio. 2014;5:e01135-14.

64. Qian J, Hospodsky D, Yamamoto N, Nazaroff WW, Peccia J. Size-resolved emission rates of airborne bacteria and fungi in an occupied classroom. Indoor Air. 2012;22:339–351.

65. Robertson CE, Baumgartner LK, Harris JK, Peterson KL, Stevens MJ, Frank DN, et al. Culture-independent analysis of aerosol microbiology in a metropolitan subway system. Appl Environ Microbiol. 2013;79:3485–3493.

66. Weinmaier T, Probst AJ, La Duc MT, Ciobanu D, Cheng JF, Ivanova N, et al. A viability-linked metagenomic analysis of cleanroom environments: eukarya, prokaryotes, and viruses. Microbiome. 2015;3:62.

67. Wood M, Gibbons SM, Lax S, Eshoo-Anton TW, Owens SM, Kennedy S, et al. Athletic equipment microbiota are shaped by interactions with human skin. Microbiome. 2015;3:25.

68. Yooseph S, Andrews-Pfannkoch C, Tenney A, McQuaid J, Williamson S, Thiagarajan M, et al. A metagenomic framework for the study of airborne microbial communities. PLoS ONE. 2013;8:e81862.

69. Handorean A, Robertson CE, Harris JK, Frank D, Hull N, Kotter C, et al. Microbial aerosol liberation from soiled textiles isolated during routine residuals handling in a modern health care setting. Microbiome. 2015;3:72.

70. Vaishampayan P, Probst AJ, La Duc MT, Bargoma E, Benardini JN, Andersen GL, et al. New perspectives on viable microbial communities in low-biomass cleanroom environments. ISME J. 2013;7:312–324.

71. Gordon J, Gandhi P, Shekhawat G, Frazier A, Hampton-Marcell J, Gilbert JA. A simple novel device for air sampling by electrokinetic capture. Microbiome. 2015;3:79.

72. Shin H, Pei Z, Martinez KA, Rivera-Vinas JI, Mendez K, Cavallin H, et al. The first microbial environment of infants born by C-section: the operating room microbes. Microbiome. 2015;3:59.
